# Valorization of Extracted Bark for Particleboard Production: A Life-Cycle Impact Assessment

**DOI:** 10.3390/polym17070925

**Published:** 2025-03-28

**Authors:** Marco Morandini, Marius Cătălin Barbu, Rozália Váňová, Stefan Kain, Jan Tippner, Alexander Petutschnigg, Lubos Kristak, Günther Kain, Thomas Sepperer, Thomas Schnabel

**Affiliations:** 1 Department Green Engineering and Circular Design, Salzburg University of Applied Sciences, Markt 136a, 5431 Kuchl, Austriamarius.barbu@fh-salzburg.ac.at (M.C.B.); stefan.kain@fh-salzburg.ac.at (S.K.); alexander.petutschnigg@fh-salzburg.ac.at (A.P.); guenther.kain@gmx.at (G.K.); thomas.schnabel@fh-salzburg.ac.at (T.S.); 2 Salzburg Center for Smart Materials, c/o Department of Chemistry and Physics of Materials, Paris Lodron University of Salzburg, Jakob-Haringer-Strasse 2a, 5020 Salzburg, Austria; 3 Department of Wood Science and Technology, Mendel University, Zemědělská 3, 61300 Brno, Czech Republic; jan.tippner@gmail.com; 4 Faculty for Furniture Design and Wood Engineering, Transilvania University of Brasov, B-dul. Eroilor nr. 29, 500036 Brasov, Romania; 5 Department of Wooden Constructions, Technical University in Zvolen, T. G. Masaryka 24, 96001 Zvolen, Slovakia; xvanova@is.tuzvo.sk; 6 Department of Physics, Electrical Engineering and Applied Mechanics, Technical University in Zvolen, 96001 Zvolen, Slovakia; kristak@tuzvo.sk; 7 Department for Wood Restoration Technology, Higher Technical College Hallstatt, Lahnstraße 69, 4830 Hallstatt, Austria

**Keywords:** bark, spruce, larch, beech, phenolic content, material flow analysis, LCIA

## Abstract

The enhanced use of wood residues from the timber industry contributes to mitigating the global climate crisis. Currently, bark, a by-product of the timber industry, is primarily burned for thermal energy generation. However, with the growing demand for lignocellulosic products and the emphasis on extending life cycles, it would be more beneficial to prioritize substantial uses of bark over thermal utilization. Although numerous methods for substantial bark utilization have been explored, a significant untapped potential remains. The extractives obtained through water extraction, for instance, can be applied to various further uses like biopolymers or medical applications. This study investigates the impact of hot water extraction on the mechanical and physical properties of bark-based panels, with the aim of extending the life cycle of tree bark and its valorization in bio-based composites. The findings demonstrate that hot water extraction can enhance the bending properties (modulus of rupture, modulus of elasticity) of bark-based panels. Additionally, the extractives obtained from the process have potential applications in the pharmaceutical and adhesive industries. The study also includes an LCIA that highlights the differences between the three scenarios addressed in this research, namely energy generation from bark-based biomass, extraction of bark, and use of extracted bark residues in the production of bark-based particleboard.

## 1. Introduction

Tree bark, a by-product of the wood-processing industry, holds remarkable potential beyond its initial protective role [1]. Some negative environmental aspects of using bark as biomass for heat or energy generation are related to increased emissions, mostly carbon monoxide and particulate matter. Additionally, an increased bark amount correlates with higher ash production [2]. Due to its ash content, tree bark was perceived as an undesirable feedstock in the past considering heightened pollution risks, material buildup (deposits of combustion residue), and potential harm to combustion equipment. A better utilization of bark and forest residues has transformed what was once a costly waste into a valuable resource. Nowadays, bark serves as a significant source of energy production. It is used in the wood processing industry, pulp and paper mills, to fuel boilers, cogeneration in biomass power plants, as well as pellet plants [3,4,5]. The chemical compounds of tree bark [6], including polyphenols, tannins, and essential oils contribute to its unique properties. Their exact properties depend on tree species, age, environmental conditions, and season [7]. These substances have been studied for their potential applications in medicine, cosmetics, and as natural preservatives, showcasing the rich biochemistry present in tree bark [8,9] and as a valuable raw material for various applications.

In general, the proportion of bark is 10% of the volume of a stem (depending on wood species, age, etc.) [10,11]. Worldwide between 0.16 and 0.19 billion cubic meters of bark are produced annually [12]. In 2018, 217 million tons of bark were harvested in the European Union. Kain et al. studied the potential use of bark as insulation material and showed some innovative properties of the bark concerning heat storage and thermal conductivity [13]. Bark serves as an excellent sound absorber as shown in previous studies. Tudor et al. and Yemele et al. analyzed the manufacture of particleboards with different bark amounts [14,15], Xing et al. investigated bark ‘suitability for medium-density fiberboards (MDFs) [16] and Domingos et al. studied the suitability of bark for oriented strand board (OSB) production [17]. Through the incorporation of minerals like clay or cement, the potential of bark as a raw material for the fabrication of fire-resistant panels is demonstrated [18,19]. Due to the high amount of extractives bark is a valuable resource for the pharmaceutical [20] and chemical industry [8,21]. Extraction of soluble compounds from tree bark involves boiling or steeping the bark in water to release a variety of chemical substances present within its structure [22]. Analytical techniques such as chromatography, spectroscopy, and microscopy are commonly used to study the detailed chemical composition and morphology of tree bark [23]. The presence of these compounds in hot water extracts imparts various properties to the solution. For example, sugars and starches [24,25] contribute to the sweetness or viscosity of the extract, while organic acids and other secondary metabolites may impart sourness or acidity [26]. Phenolic compounds in bark, such as tannins [22], are known for their antioxidative properties and may also exhibit antimicrobial or astringent effects. Nevertheless, the significant presence of free, glycosidic, and polymeric sugars in the raw extract can hinder its application in various uses [27]. The stilbenoids like betulin and subarin [28] of bark could be valorized by applying environmentally benign biorefinery processes, including pressurized hot water extraction or supercritical fluid extraction, followed by further purification [29].

The substances gained during hot water extraction can be used for further applications [30,31,32] due to their bioactive antiviral, antibacterial, and antioxidant properties [20,33,34]. The bark, once extracted, can undergo pyrolysis, leading to the production of biochar for market use, energy-rich gases, and a liquid fraction [35]. Nowadays, mostly in pulp mills or sometimes in the wood-processing industry, the necessity to incinerate all bark generated on-site has been eliminated, particularly during warmer seasons. Consequently, alternative methods are explored to utilize surplus bark, including supplying it to external power boilers or converting it into other energy resources or into value-added products. Employing a heated press nip could decrease the moisture content of the targeted one, as the moisture content significantly influences the heating value [36].

A process model integrating hot water extraction (HWE), slow pyrolysis, and anaerobic digestion (AD) was employed for the utilization of pine and spruce bark. Initially, tannins and polyphenols were extracted through HWE. Subsequently, the residual material underwent pyrolysis to yield biochar of marketable quality, gas for energy, and liquid fractions. Further separation of the liquid fraction yielded aqueous acidic and tar fractions. Bark, extracted bark residue, and the acidic liquid fraction from pyrolysis were then subjected to AD to generate biomethane and digestate. Methane yields from pine and spruce bark and their extracted residues were limited with marginal differences observed. Cascade processing of softwood bark can improve the performance of subsequent single processes and utilize biomass sources with higher efficiency [35].

The presence of extractives in bark affects bark-based composites in various ways, both detrimental and beneficial. Extractives can negatively influence the adhesive setting, leading to a decrease in particle–particle bond strength that significantly reduces internal bond strength. Conversely, phenolic extractives have the potential to react with formaldehyde, limiting water absorption, and enhancing thickness swelling [37].

This study investigates the mechanical and physical properties of composite boards of three types of extracted bark (spruce, larch, and beech). It proposes a holistic approach to bark processing, hot water extraction, and different applications of bark aiming at lowering greenhouse gas emissions and extending of cascading use of wood.

## 2. Materials and Methods

For this study, three types of bark species, namely spruce (*Picea abies*), larch (*Larix decidua*), and beech (*Fagus sylvestris*), provided by Deisl Sawmill (Adnet, Austria), were used. The initial moisture content of the bark was 80%. The massive bark planks were ground by means of a 4-shaft shredder RS40 at Untha Co. (Kuchl, Austria), with a mesh of 30 mm. The fraction from 4 to 10 mm was chosen for this study. Before pressing to obtain the bark-based composites, the particles of spruce, larch, and beech bark were dried to 8% moisture content at 200 to 250 mbar in a vacuum kiln dryer Brunner–Hildebrand High VAC-S, HV-S1 (Hannover, Germany). The raw material bark served first for heat generation (Scenario 1), secondly, for gaining phenol extractives (Scenario 2), and thirdly, as a secondary raw material for the manufacture of bark-based composites (Scenario 3).

**Figure 1:**
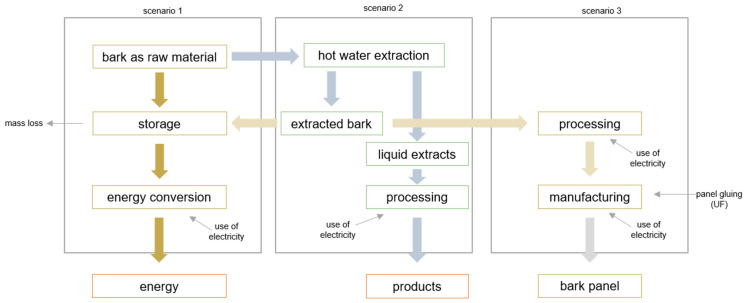
Simple process flow diagram and system boundaries for the analysis of three possible scenarios concerning superior valorization of bark.

Based on three various scenarios for the bark processing concerning the deliverable products (e.g., energy, by-products (e.g., tannin and bark panels)), the processes (Figure 1) are defined as follows:

Scenario 1:

This process describes the conventional processing of bark biomass for energy generation in power or heating plants. Normally, the tree bark is stored under the roof and is burnt moist in energy plants. The fresh bark (80% moisture content), stored outside, is subjected to abiotic and biotic effects and losses of mass [4,38]. Several studies of dry matter loss during storage reported values of 5 and 30% depending on time, conditions, and pile height [4,39]. In this study, an average dry matter loss of around 10% was used for the assessment.

Scenario 2:

This scenario describes the use of fresh bark as a source of hot-water-soluble extractives. This extracted bark can be used as fuel for generating power and/or thermal energy. Furthermore, there are two possible options to use the extracted bark. On one hand, the wet bark can be stored outside to reduce the moisture content. On the other hand, the extracted bark can be machine-dried before the material is burned. These different process steps influence the environmental impacts in Scenario 2 and also the recovered bark extracts could impact the economic view of this additional process with the input of thermal energy.

Scenario 3:

The last scenario deals with the innovative approach to using the bark as raw materials for different applications and added-value products. The fresh bark is extracted with a hot water extraction process (Scenario 2). The extracts are dried to provide a powder for further usage. Moreover, the extracted bark materials are dried and prepared for manufacturing of bark panels with a standard urea formaldehyde glue for wood-based panels. In the end, Scenario 3 could offer a new business model for bark panels and bark extractives.

The functional unit (FU) for Scenario 3 is a 1 m^2^ bark-based board from the bark with 8% moisture content, bonded with 8% UF adhesive.

The system was selected to study the environmental impacts of multi-level processing of bark particles and compare different scenarios. The values of mass and energy balances for the hot water extraction, bark press, and drying as well as energy conversion and manufacturing processes were taken from experimental data and literature. Table 1 presents the operating values used for this study.

**Figure 2:**
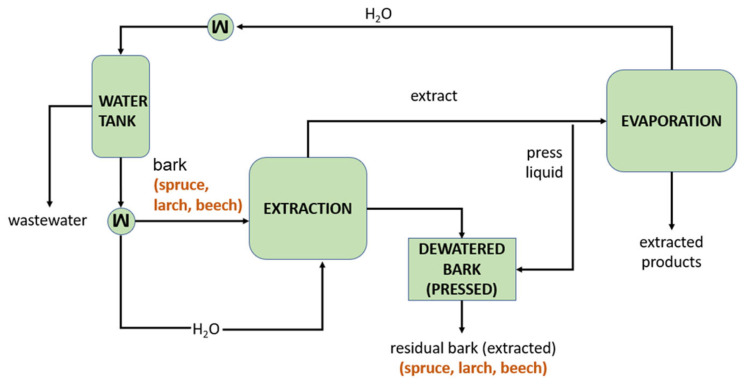
Hot water extraction process of tree bark, with dewatering of extracted bark for further added-value applications.

The hot water extraction (HWE) process (Figure 2) was conducted in a batch mode within a stirred tank reactor. Given the low amount of water in the initial bark feed, a purge stream of water was required, which was subsequently separated and treated as wastewater. Through internal heat recovery, the net heat demand was decreased by 60%. The extracted bark was consequently dewatered in a hydraulic press type Höfer HLOP 280, to achieve a moisture content of 25%.

Spruce, larch, and beech bark with a particle size of 4 to 10 mm were used for HWE. For the chemical extraction sodium bisulfite NaHSO_3_ (Carl Roth, Karlsruhe, Germany) was used. Analytical grade reagents were employed for the colorimetric assays (total phenolic content (TPC)) and for the antioxidant activity 2,2-diphenyl-1-picrylhydrazyl (DPPH), acquired either from VWR or Carl Roth.

HWE was carried out in a glass reactor (20 L) equipped with a heating mantel, temperature control, and stirring system. The solid to liquid ratio was fixed at 1:7 (bark–water). Extraction was assisted with 3 wt.% sodium bisulfite based on the dry weight of the bark to increase the solubility of tannins and other extractives [23,43]. After the set extraction time (2 and 4 h), heating and stirring were switched off, and the mixture was allowed to cool down a little. Afterwards, the spent bark particles were separated from the tannin-enriched liquor and processed separately.

Extract processing: The liquid extract was filtered through glass filters with decreasing pore size (down to 40 µm) to remove fine bark particles and dust. Afterwards, an aliquot was used to determine the solid content of the liquor, thus allowing it to calculate extraction yield. Furthermore, some of the liquid extract was dried using a rotary evaporator at 72 mbar and 60 °C water bath temperature to obtain a dry, and powdered extract.

Total phenolic content: The bark extracts were characterized for their total phenolic content (TPC) and their antioxidative activity. For the estimation of TPC, the well-established method of Folin and Ciocalteu was used as described previously [23,44]. In short, 200 µL of the aqueous extract solution (concentration 1 mg mL^−1^) was diluted with 3 mL of deionized water and mixed with 500 µL of Folin–Ciocalteu phenol reagent and incubated for 3 min. Afterward, the pH was raised with 2 mL of 20% sodium carbonate solution to precipitate the colored complexes. Adsorption at 745 nm was measured after a 1 h incubation period. TPC was expressed as mgGAE g^−1^ (GAE = Gallic acid equivalent). A calibration curve was created by stepwise dilution of a 1 mg mL^−1^ aqueous gallic acid solution into 500, 200, 100, and 50 µg mL^−1^. R^2^ of the calibration curve was 0.9998.

To estimate the antioxidative properties of the bark extracts, a radical scavenging assay based on the free stable radical 2,2-diphenyl-1-picrylhydrazyl (DPPH) was employed. A total of 30 µL of aqueous extracts (1 mg mL^−1^) were mixed with 3 mL methanolic DPPH solution (6 × 10^−5^ M) and incubated in the dark for 15 min. Absorbance was measured at 515 nm and inhibition-% was calculated according to Equation (1), where *A*_0_ describes the initial absorbance of DPPH solution and *A*_15_ describes the absorbance of the samples after 15 min incubation. For all colorimetric measurements, a UV/Vis spectrophotometer VWR P4 was used and performed in triplicate.

(1) $$Inhibition\;\%=\frac{A_{0}-A_{15}}{A_{0}}$$

The extracted bark served as a secondary raw material for the manufacture of bark-based particleboards. The bark particles were mixed with 8% of adhesive (UF-adhesive type 10F102 (Dynea, Krems, Austria)) for 2 min at roughly 200 rpm using a ploughshare mixer. Resin-coated particles were subsequently discharged and spread out evenly in a 100 × 100 cm mold and manually pre-compressed. The panels were then pressed with a hydraulic press Höfer HLOP 280 (Taiskirchen, Austria) for 6 min, at a temperature of 180 °C and a fixed thickness of 8 mm. For each type of bark, three panels were produced.

According to the given scenarios presented in Figure 1, a process-oriented life-cycle impact assessment (LCIA) was conducted using SimaPro Analyst 9.6.0.1. All scenarios were modeled through the ecoinvent 3.10 cut-off database and analyzed by EN 15804 + A2 [45] calculation method encompassing impact categories of climate change (biogenic, fossil, land use, and total), acidification, eutrophication (terrestrial, marine, freshwater), ozone depletion, photochemical ozone formation, resource use (fossils; minerals and metals), and water use. The corresponding amount of bark for the targeted functional unit was chosen to be 9.36 kg of fresh bark with a moisture content of 80%, which corresponds to 1 m^2^ of bark-based particleboard in Scenario 3. The processes under study cover the initial input of bark and other materials and energy and end with the creation of 1 m^2^ of the board. Primary data based on actual calculations during the processing of the boards were used in the assessment. System flows are given in the schemes for all three scenarios. Transportation is neglected due to laboratory conditions which is also a limitation of the study. The results could give feedback to manufacturers in early-stage decision-making activities to understand the actual production impacts of the board itself by varying the process.

## 3. Results and Discussion

### 3.1. Hot Water Extraction of Tree Bark

The first part of the results of this study presents the total phenolic content, antioxidant activity, and extraction yield, determined after the hot water extraction process of spruce, larch, and beech bark (Table 2). For all tree bark species, the total solid content and the extraction yield increased when the extraction took longer (4 h). As a measure for the phenolic content of the extracts, results of the Folin–Ciocalteau assay (TPC) are presented as milligram gallic acid equivalent per gram extract (mgGAE/g). Generally, a higher TPC suggests a larger proportion of the extract to consist of tannins, phenolic acids, and other reducing molecules.

In the case of spruce bark, for a 2-h extraction process, the lowest yield for spruce bark was a solid compound of 31.0 g/kg. When the extraction time was doubled to 4 h the total solid content increased to 71.3 g/kg (7.13%). Lacoste et al. (2015) reported an extraction yield of 14.5% after 2 h of hot water extraction of Norway spruce [46]. The higher yield could be explained by different solid–liquid ratios, variations in the extraction setup, and freshness/origin of the bark.

For the larch bark, the highest extraction yield after 2 h was 13.7% (137.1 g/kg). The highest yield was observed after 4 h with 21.19% and a total solid content of 211.9 g/kg, respectively. Hot water extraction processes for larch bark were analyzed in other research studies. Refs. [47,48] mentioned in their works an extraction yield between 91.7 g/kg and 103.6 g/kg. The differences between the present study and the results from the literature can be justified by methodological variations (e.g., extraction temperature, pressure, and iterations) as well as natural differences from tree variations (e.g., origin and age of the tree, harvesting season).

The analysis of extracts from beech bark revealed an increase from 10.34% to 30.91%, which means 3-fold higher, after 4 h water extraction. For spruce bark, the yield after 4 h HWE was 2.3-fold and for larch bark, 1.55-fold, compared to the extraction for two hours.

In terms of TPC and antioxidative potential, 4 h larch bark extract shows the most interesting results. With almost 460 mgGAE/mg, TPC is nearly three times the phenolic content of 2 h spruce bark extract. For the two coniferous species, an increase in extraction time has a positive influence on the phenolic content and the antioxidative inhibition. Reddy et al. (2010) reported a strong correlation between phenolic content and radical scavenging potential (DPPH), which was confirmed in our study of larch and spruce bark extracts [49]. On the other hand, beech bark extract behaved in a different fashion. A longer extraction time, despite increasing the antioxidative potential (as indicated by the higher DPPH inhibition), resulted in a lower concentration of phenolics and other reducing substances. One possible explanation might be the uncertainty of the FC assay, as it is not only reacting with phenolics, but also reducing sugars [50]. Additionally, Ref. [51] reported decreasing phenolic yield for increased reaction time in some water-based extraction systems for beech bark.

**Figure 3:**
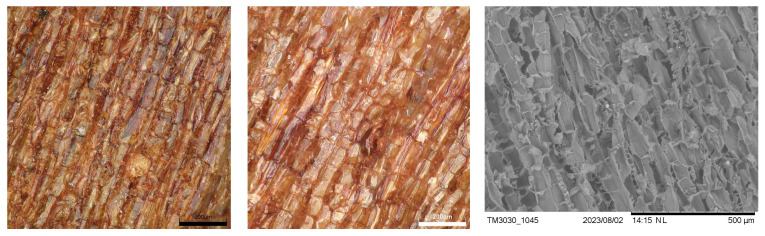
Micrographs of untreated (**left**) and extracted larch bark (**middle**). SEM micrograph of extracted larch bark (**right**).

Additionally, as a representative for the three barks used, micrographs of larch bark before and after extraction, as well as an SEM micrograph of the extracted bark are displayed in Figure 3.

### 3.2. Physical and Mechanical Properties of the Bark-Based Panels

The ANOVA results are detailed in Table 3, listing the factors that statistically significantly influenced the panel properties. The statistical model for the dependent variables (IB, MOR, MOE, TS, WA, and SW) was highly significant (*p* < 0.001) for all variables. The explanatory power, indicating the strength of the relationship between the dependent and independent variables, was relatively low, with *η*^2^ values not higher than 0.6 for all investigated panel properties.

The results of the physical properties (density and thickness swelling) and mechanical properties—internal bond (IB), modulus of rupture (MOR), modulus of elasticity (MOE), and screw withdrawal resistance on surface (SW) are presented in Table 4, with standard deviation (SD) in parentheses.

**Figure 4:**
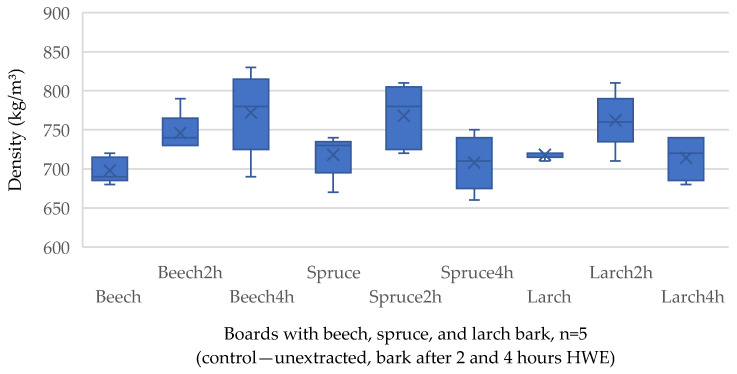
Density of the bark-based panels produced with unextracted and hot water-extracted raw materials. × marking the average value.

The target density (Figure 4) of all bark-based panels is 750 kg/m^3^. Due to the manual forming of the press mattress, the measured density ranges between 680 and 830 kg/m^3^. As results from Table 2, given the *p*-values of 0.16 and 0.17, which reflect no significant effect of bark species and steam exposure, a trend that reflects differences between the groups (untreated, bark after 2 and 4 h HWE), cannot be drawn.

**Figure 5:**
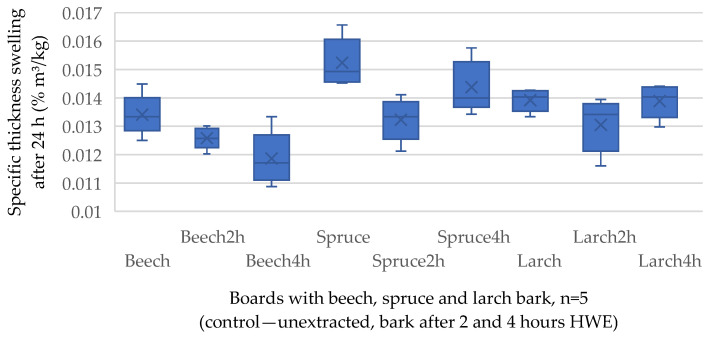
Specific thickness swelling of the bark-based panels produced with unextracted and hot water-extracted raw materials. × marking the average value.

Table 2 indicates no significant effect of the bark species on the thickness swelling at a 0.01 probability level. Considering TS (Figure 5), no interactions were observed between bark species (0.1) and steam exposure time (0.2). The average value of TS is 0.02% × m^3^/kg for all groups, which is comparable with the values determined in [37], which dealt with panels made of extracted black spruce and trembling aspen bark. In this study, the specific TS ranged from 0.012 to 0.038% × m^3^/kg, compared to this study, where a minimal TS of 0.012% × m^3^/kg and a maximal level of 0.016% × m^3^/kg were achieved. The lowest TS achieved by all groups made with beech bark can be due to the highest amount of lignin in the chemical composition of this bark species (34%), as reported by [52]. Lignin is hydrophobic, reduces water permeability, and contributes to the overall durability and longevity of lignocellulosic materials, increasing their resistance to fungal and microbial attacks [53]. The value associated with the benchmark (P1 grade, for general-purpose boards for use in dry conditions, according to EN 317:2005 [54]) of 0.01% × m^3^/kg was reached by most of the tested bark-based boards.

The statistical model for the dependent variables IB, MOR, and MOE (Table 3) was highly significant, reflected in a probability level of 0.001.

**Figure 6:**
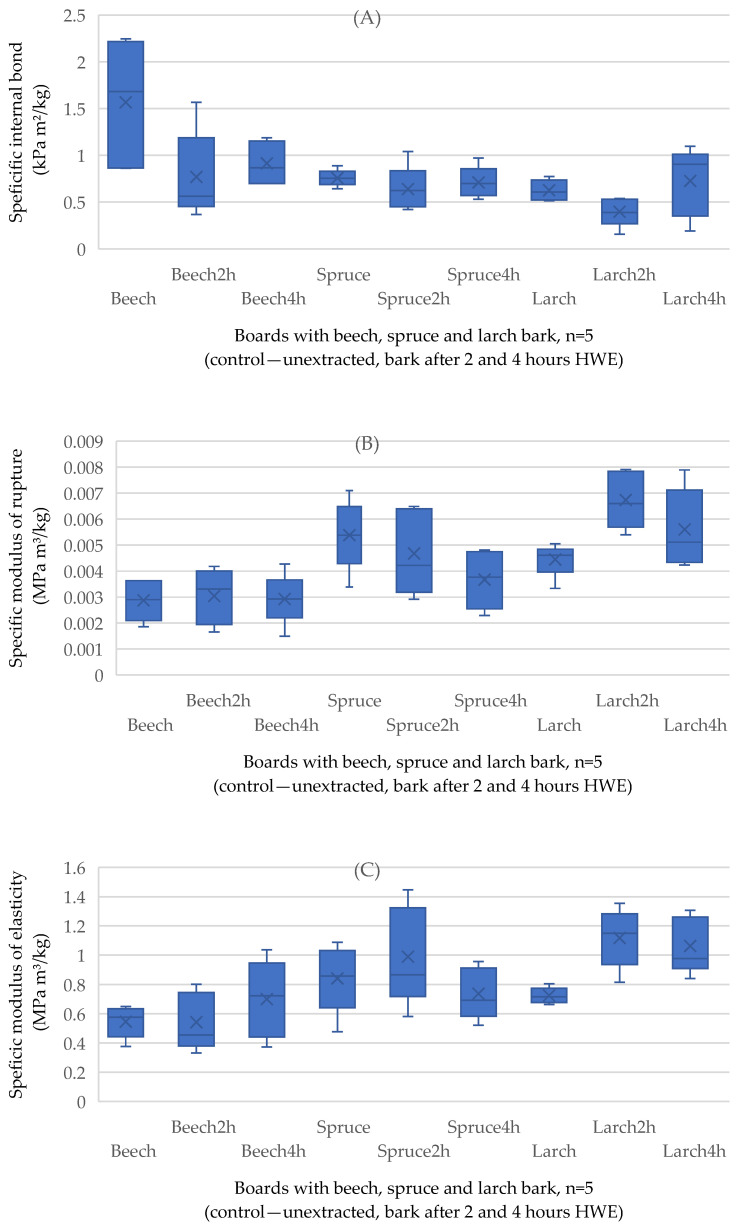
Specific internal bond of the bark-based panels produced with unextracted and hot water-extracted raw materials (**A**); specific modulus of rupture of the extracted bark-based panels (n = 5) (**B**) and specific modulus of elasticity of the extracted bark-based panels (n = 5) (**C**). × marking the average value.

The highest levels of specific internal bonds (Figure 6A) were measured for the panels made of beech bark. A trend cannot be determined also in this case, because as density increases, internal bond decreases for certain panels (e.g., the panel with bark beech after 4 h HWE) and is not directly correlated with the density (R^2^ = 0.5). The effect of HWE on IB could be explained as a decrease in pH value, along with a reduction in reactive materials, such as polyphenols, particularly bark tannin, which can positively interact with the adhesive and may have been extracted during the hot water treatment [37]. The value for the benchmark (P1) EN 312:2010 [55] was reached only by some samples from the panel made with untreated beech bark, because the mean IB in this case is 1.56 kPa m^2^/kg. For the rest of the panels, with untreated and extracted bark, the internal bond was under the benchmark, but still higher compared to the results of the finding of [37], who determined values up to 0.7 kPa m^2^/kg for similar panels with spruce and trembling aspen bark.

The influence of bark species is interesting considering the bending properties of bark-based composites, because the beech bark showed the weakest bending properties (Figure 6B,C), despite the highest amount of cellulose (37%) in its chemical composition [53]. Due to its degree of polymerization and linear orientation, cellulose is responsible for the strength of lignocellulosic fibers [56]. MOR and MOE EN 312:2010 for beech-bark panels were the lowest of all groups, which could be caused by reduced tack of the bark and the rate of heat transfer through bark particle composite. This can be explained by incomplete adhesive curing, which causes a decrease in MOE and MOR [37]. None of the tested boards reached the values for P1 grades (0.0325 MPa m^3^/kg for MOR and 4.8 Mpa m^3^/kg for MOE). The explanatory power of 0.06 and 0.1 between the steam exposure time and MOR and MOE, respectively, demonstrate their weak dependence. Results from a similar study conducted with black spruce and trembling aspen bark revealed a similar tendency, of improved bending properties when the bark particles were extracted with hot water [37].

### 3.3. Holistic Assessment of Different Processing Scenarios

**Figure 7:**
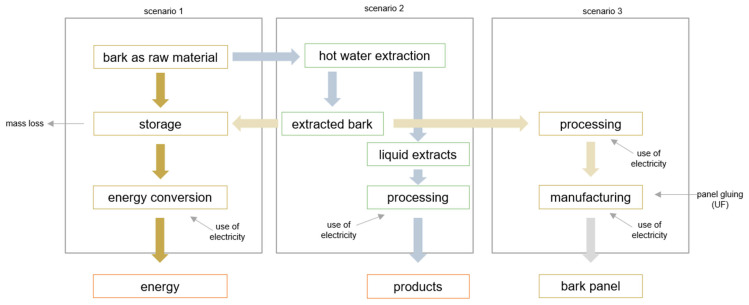
Different scenarios for the valorization of raw material bark.

The functional unit (FU) was 1 m^2^ of a dry bark panel with a thickness of 8 mm and a target density of 750 kg/m^3^. An overview of the mass and energy flows for producing a spruce bark panel (1 FU) is shown in Figure 7. In this connection, the spruce bark particles were extracted for two hours and finally processed into the bark panel at laboratory-scale.

**Figure 8:**
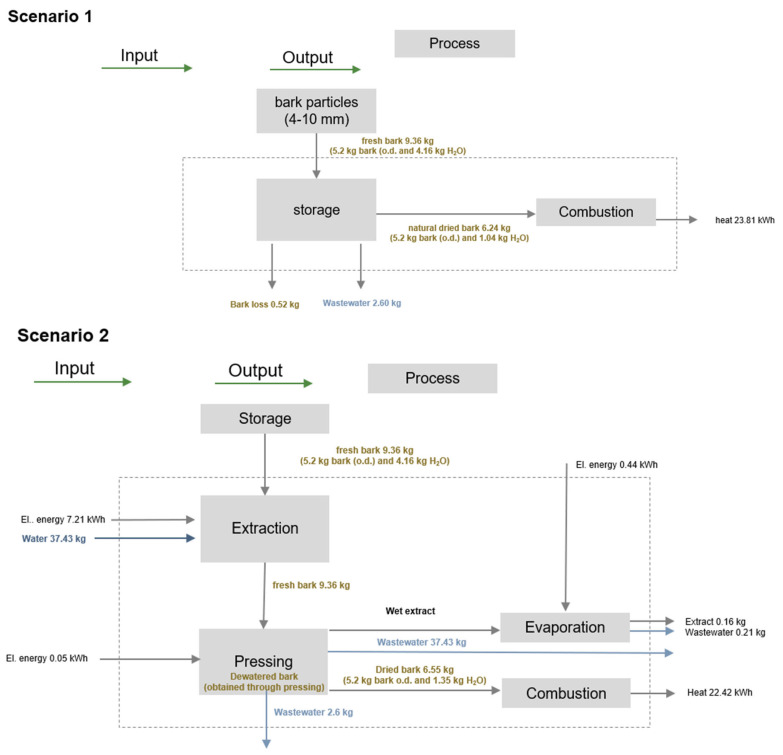

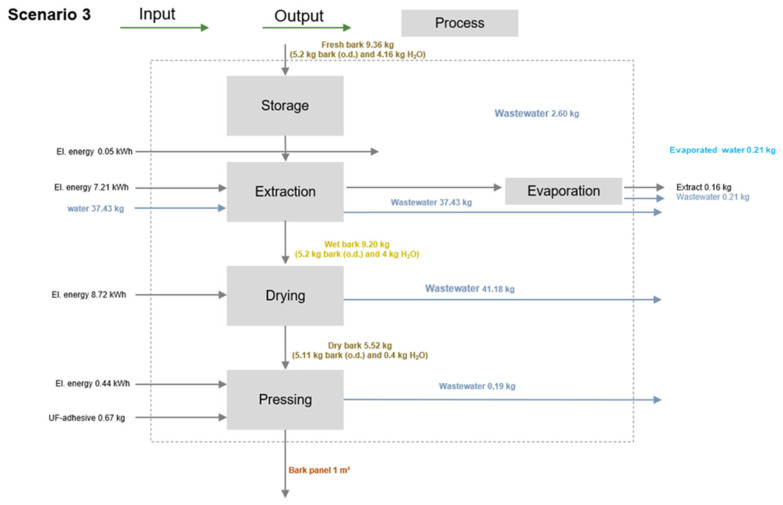
Scenario 1, involving energy generation from bark biomass; Scenario 2, involving hot water extraction of the bark and subsequently the energy generation from bark biomass; and Scenario 3, involving hot water extraction and the manufacturing of bark-based particleboards.

These process diagrams (Figure 8) present three possible scenarios regarding further processing of bark. In Scenario 1, the bark is stored outdoors up to a material moisture content of 20% and then fed into the combustion process. In Scenario 2, another process step is added, namely the hot water extraction. Scenario 3 is the most complex one, because the bark (untreated, extracted after 2 h, and extracted after 4 h) is the raw material for manufacturing bark-based particleboards. In the process diagrams (Figure 8), the electrical energy for the pressing process was calculated for the heating-up of the bark material from 20 to 180 °C according to [42].

The results in Figure 8 (Scenario 3) are based on the findings of this study and lab-scale data collected from the literature on extracting phenolic compounds and processing bio-based materials (e.g., wood chips). Many of the current studies were focused on the LCA comparison regarding the environmental impact of the different extraction methods (e.g., hot water or ultrasound-assisted extraction) [57,58,59].

#### Life-Cycle Impact Assessment

Table 5 presents the general environmental profiles of the scenarios presented in Figure 8, revealing that the hot water extraction, followed by the manufacturing of the bark-based composites determined the highest impacts across all categories, while the energy generation from natural dried fresh bark exhibited the lowest impacts. If the total impact of climate change is scrutinized, it results that the value of −1.516 kg CO_2_ eq calculated for Scenario 1 is 13-fold higher compared to Scenario 2 and 2.7-fold lesser than that of the value for Scenario 3. The energy consumption is 5 times higher for Scenario 2 and 12.2 times higher for Scenario 3, when compared to Scenario 1. Assessing these results with other studies is challenging due to variations in LCIA study parameters (such as functional units, system boundaries, product allocation, and impact assessment methods).

The high energy consumption is related to the technological steps involved in the scenarios. Analogous results were also obtained in previous research [28,57,60]. While Scenario 1 shows the lowest negative environmental impact in all categories, it is necessary to realize the simplicity of this process.

Regarding all three scenarios, the second one could be a compromise for settling the environmental impacts and the bark usage. However, utilization of bark for extraction purposes depending on the extraction process used can be less or more burdensome for the environment. Bark extraction methods are covered by Carlqvist et al. [58] and Ding et.al [59], proving different types of extraction processes report various impacts related to the additives used.

Since Scenario 3 covers the process of particleboard manufacturing, the range of impacts remains legitimate. Each technological step carries a certain burden within its life cycle. Despite the highest impact of Scenario 3 in nearly all categories, particleboard production remains a promising way of bark usage primarily when circular economy principles are coming forward. According to Gavioli et al. [61], it is also possible to reduce CO_2_ emissions from particleboard production by up to 51% using appropriate additives. In addition, Yılmaz [62] states that UF adhesive and electricity consumption are the most significant contributors to resource depletion.

## 4. Conclusions

This study has demonstrated that tree bark, in addition to being primarily used for energy generation (Scenario 1), can also serve as a source of phenolic compounds when extracted with hot water (Scenario 2). Subsequently, the extracted bark can be utilized as raw material for the production of particleboards (Scenario 3). After 2 and 4 h of hot water extraction, the yield of phenolic content and antioxidant activity increases, yielding extracted chemicals potentially useful in applications such as the pharmaceutical industry or the production of green composites. In terms of mechanical properties, the bark-based particleboards met the requirements only for specific thickness swelling (0.01% × m^3^/kg). For specific internal bonds, only untreated beech bark reached the requirements with 1.56 kPa m^2^/kg. For bending properties, particleboards made solely from extracted bark did not meet the requirements for specific MOR (0.0325 MPa m^3^/kg); the closest was 2 h extracted larch bark with 0.006 kPa m^3^/kg. For specific MOE, the highest values were again achieved by 2 h larch with 1.11 MPa m^3^/kg, but still only reached a quarter of the required strength. Although the bark-based particleboards with enhanced properties did not meet the requirements for P1 grade particleboard, it is suggested that with an optimized ratio of bark and wood, the internal bond, bending strength, and modulus of elasticity could be improved. Mass production of tree bark for particleboard manufacturing relies on efficient debarking techniques, followed by cleaning and size reduction for processing. Careful selection of tree bark species ensures optimal performance of the final product. This large-scale operation supports the production of environmentally friendly materials. The resulting particleboard can constitute a sustainable alternative to traditional particleboards.

The life cycle inventory analysis conducted in this study highlights the complexity of the three scenarios in assessing the environmental impact of bark biomass.

## Figures and Tables

**Table 1:** Collected energy consumption/generation data of the different processes for superior use of tree bark.

**Operation**	**Value**	**Unit**	Reference
Natural mass loss due to storage	10	%	[4]
Hot water extraction—2 h	0.77	kWh/kg	Present study
Hot water extraction—4 h	0.921	kWh/kg	Present study
Evaporation	0.01167	kWh/kg	[40]
Pressing for remove water	0.005	kWh/kg water	[40]
Bark drying (from 30 to 8% moisture)	1.5	kWh/kg water	[41]
Heating up for panel pressing	0.08029	kWh/kg	[42]
Dry calorific value for spruce bark	20.668	MJ/kg	Present study
Dry calorific value for larch bark	19.822	MJ/kg	Present study
Dry calorific value for beech bark	16.321	MJ/kg	Present study

**Table 2:** Average values for total phenolic content, antioxidant activity of the various oven-dried bark extracts, and extraction yield of oven-dried bark after 2 and 4 h HWE.

Sample	Total Phenolic Content [mgGAE/g]	Antioxidant Activity [Inhibition %]	Yield [%]
Spruce bark after 2 h HWE	160.16 *	27.55 *	3.10
Spruce bark after 4 h HWE	202.32 *	44.08 *	7.13
Larch bark after 2 h HWE	324.04 *	53.16 *	13.71
Larch bark after 4 h HWE	458.21 *	82.16 *	21.19
Beech bark after 2 h HWE	317.65 *	62.30 *	10.34
Beech bark after 4 h HWE	231.84 *	76.76 *	30.91

* mean value of 4 samples.

**Table 3:** Results of the ANOVA with *p*-values and $\eta^{2}$-values for the explanatory variables.

	Density		IB		MOR		MOE		TS	
	*p*	$\eta^{2}$	*p*	$\eta^{2}$	*p*	$\eta^{2}$	*p*	$\eta^{2}$	*p*	$\eta^{2}$
Model	0.001	0.46	0.001	0.49	0.001	0.57	0.001	0.49	0.001	0.35
Bark species	0.16	0.61	0.001	0.27	0.001	0.38	0.001	0.25	0.101	0.17
Steam exposure	0.17	0.24	0.27	0.11	0.19	0.06	0.17	0.1	0.2	0.03

**Table 4:** Effect of steam exposure time (0, 2, and 4 h) of the specific mechanical properties of the bark-based boards (IB = internal bond, MOR = modulus of rupture (N/mm^2^), MOE = modulus of elasticity (N/mm^2^), TS = thickness swelling (%)). Standard deviation is reported in parentheses.

Board Type	Density (kg/m^3^)	Specific TS (%)	Specific IB (N/mm^2^)	Specific MOR (N/mm^2^)	Specific MOE (N/mm^2^)
Beech bark untreated	698 (15)	0.01 (0.0007)	0.001 (0.0006)	0.002 (0.0007)	0.54 (0.09)
Beech bark after 2 h HWE	746 (22)	0.01 (0.001)	0.0007 (0.0004)	0.003 (0.001)	0.54 (0.17)
Beech bark after 4 h HWE	772 (47)	0.01 (0.001)	0.0009 (0.0002)	0.002 (0.0008)	0.69 (0.23)
Spruce bark untreated	718 (25)	0.01 (0.001)	0.0007 (0.0006)	0.005 (0.001)	0.84 (0.2)
Spruce bark after 2 h HWE	768 (37)	0.01 (0.001)	0.0006 (0.0001)	0.04 (0.001)	0.98 (0.3)
Spruce bark after 4 h HWE	708 (31)	0.01 (0.0008)	0.0007 (0.0001)	0.003 (0.001)	0.73 (0.15)
Larch bark untreated	724 (6)	0.01 (0.0008)	0.006 (0.0009)	0.004 (0.0005)	0.72 (0.04)
Larch bark after 2 h HWE	762 (32)	0.01 (0.001)	0.0006 (0.0001)	0.006 (0.001)	1.11 (0.17)
Larch bark after 4 h HWE	714 (25)	0.01 (0.001)	0.0007 (0.0003)	0.005 (0.001)	1.06 (0.17)

**Table 5:** Results of life-cycle impact assessment for 3 scenarios involving the use of bark.

Impact Category	Unit	Scenario 1	Scenario 2	Scenario 3
Acidification	mmol H^+^ eq	8.630	14.294	18.349
Climate change—total	kg CO_2_ eq	−1.516	−0.113	−4.038
Climate change—biogenic	kg CO_2_ eq	−2.112	−2.328	−8.797
Climate change—fossil	kg CO_2_ eq	0.595	2.211	4.753
Climate change—LULUC	g CO_2_ eq	1.206	3.318	5.782
Eutrophication, marine	g N eq	3.365	4.344	3.691
Eutrophication, freshwater	g P eq	0.260	1.966	3.998
Eutrophication, terrestrial	mol N eq	0.038	0.046	0.036
Ozone depletion	mg CFC-11 eq	0.004	0.045	0.148
Photochemical ozone formation	kg NMVOC eq	0.011	0.014	0.014
Resource use, fossils	MJ	7.078	33.166	86.259
Resource use, minerals, and metals	mg Sb eq	3.367	34.076	80.924
Water use	m^3^ depriv.	0.283	0.446	2.059

## Data Availability

The original contributions presented in this study are included in the article. Further inquiries can be directed to the corresponding author.
